# The concurrent association of inflammatory polymyositis and Crohn’s ileo-colitis in a Sri Lankan man: a case report of a rare association and literature review

**DOI:** 10.1186/1471-230X-14-35

**Published:** 2014-02-20

**Authors:** Vipula R Bataduwaarachchi, Nilesh Fenandopulle, Upul Liyanage, Champa Jayasundara

**Affiliations:** 1 Department of Medicine, National Hospital, Colombo, Sri Lanka; 2 Department of Gastroenterology, National Hospital, Colombo, Sri Lanka; 3 Department of Medicine, Sri Jayawardanapura General Hospital, Colombo, Sri Lanka

**Keywords:** Inflammatory polymyositis, Crohn’s disease, Rhabdomyolysis, Ileocolitis

## Abstract

**Background:**

Crohn’s disease is a relapsing, systemic inflammatory disease affecting the gastrointestinal tract with associated extraintestinal manifestations and immune disorders. Among the few cases reported, the association of Crohn’s disease with polymyositis varies in its complexity and severity. We report here the first known case of inflammatory polymyositis leading to rhabdomyolysis in a male patient diagnosed with Crohn’s ileocolitis.

**Case presentation:**

A 42-year-old previously healthy man presented with acute polymyositis leading to rhabdomyolysis. The acute nature of the illness raised the suspicion of an infective, toxic, or metabolic insult, which was excluded during further investigations. Prolonged low-grade fever and raised inflammatory markers led to the suspicion of inflammatory polymyositis, which was confirmed by electromyography and muscle histology. In the absence of an infective cause, the concurrent association of prolonged diarrhea containing blood and mucous after recovery from an acute phase of myositis proved a diagnostic challenge. Ileocolonoscopy findings of extensive aphthous ulceration with skip lesions extending to the terminal ileum, and histology showing polymorph infiltration of the lamina propria, transmural involvement, and micro abscess formation was suggestive of Crohn’s disease. Sensory motor axonal peripheral neuropathy, which is another rare association of inflammatory bowel disease, was also present.

**Conclusion:**

An unrecognized genetic predisposition or altered gut permeability causing disruption of the gut immune barrier triggering an immune response against skeletal muscles may have contributed to this unique association. Both polymyositis and Crohn’s ileocolitis responded well to corticosteroids and azathioprine, which is supportive of their immune pathogenesis. Myositis can be considered to be a rare extraintestinal manifestation of Crohn’s disease and can be used in the differential diagnosis of corticosteroid or hypokalemia-induced myopathy in Crohn’s disease.

## Background

Crohn’s disease (CD) is a relapsing systemic inflammatory disease that predominantly affects the gastrointestinal tract. Its association with extraintestinal manifestations is complex, causing many diagnostic and management challenges. Seronegative spondyloarthropathies have been reported in 33% of CD patients [1], while erythema nodosum is more common in CD than pyoderma gangrenosum [2]. Episcleritis, scleritis, and uveitis occur in approximately 3% of patients with inflammatory bowel disease (IBD) and are more often seen in patients with ulcerative colitis than CD [3,4]. Primary sclerosing cholangitis, thromboembolic events, and nephrolithiasis are less common associations of IBD. Moreover, peripheral neuropathy is not well characterized in IBD and its incidence varies from 0.9 to 3.6% [5,6]. Chronic idiopathic inflammatory myopathy (IIM) is also a rare association with CD, with one known study by Szabo et al. of a female CD patient presenting with chronic IIM [7].

Few cases have reported an association of polymyositis with CD, although the occurrence of polymyositis, alopecia universalis, and primary sclerosing cholangitis was observed in a male CD patient in Germany [8], and Hall documented a case of focal myositis involving the left gastrocnemius muscle [9]. The timing of the onset of myositis has been shown to vary widely among patients with IBD [10-12]. Only a small number of cases of rhabdomyolysis in association with CD have been reported, of which the subclinical presentation is more common than the acute presentation [13]. Most of these were caused by hypokalemia associated with severe IBD, and only one case report describes probable immune pathogenesis [10,14-16]. The study of different disease associations will provide new insights into the complex pathogenesis of IBD, and expansion of the scope of extraintestinal manifestations will aid the early diagnosis and management of CD. We herein report the first known case of inflammatory polymyositis complicated with rhabdomyolysis in a male patient diagnosed with CD.

## Case presentation

A 42-year-old previously healthy man was admitted to the emergency department with sudden-onset right upper limb swelling, pain, and weakness associated with dysphagia, reduced urine output, red-colored urine, and melena. He had consumed three pints of alcohol 3 days prior to this presentation but had no history of travel, trauma, or illicit drug abuse. He had acidotic breathing on examination.

A full blood count showed severe anemia, thrombocytopenia, and neutrophil leukocytosis. The inflammatory markers, erythrocyte sedimentation rate and C-reactive protein levels were well above the normal range, which was suggestive of an inflammatory process. The full urine report confirmed myoglobulinuria. Creatine kinase levels exceded 50,000 U/L, and serum myoglobin levels were more than 60,000 ng/mL, which was suggestive of rhabdomyolysis. Clinical and biochemical parameters confirmed acute kidney injury (AKI). Aspartate transaminase levels (4,795 U/L, normal range 8–48 U/L) exceeded alanine transaminase (ALT) levels, and were 100 times the normal level, which explained the leakage from muscle damage. ALT levels were also 40 times the normal level (2,268 U/l, normal range 7–55 U/L), with associated high activated partial thromboplastin time and prothrombin time indicating simultaneous liver injury. D-dimer levels were within normal limits and disseminated intravascular coagulation was excluded. Arterial and venous Doppler scans of the upper limbs excluded thrombosis as the cause of severe edema and revealed marked subcutaneous and soft tissue edema. Initial diagnosis suggested an acute metabolic, toxic, or infective insult causing myositis and rhabdomyolysis leading to AKI.

**Figure 1:**
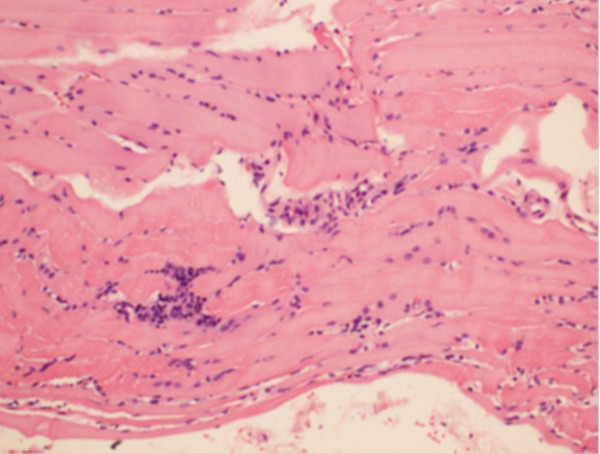
**Section of muscle biopsy stained with HE.** Muscle fibers show perimiceal inflammation, unequal sizes, and swelling. Mononuclear infiltrations of inter-fiber areas are seen. There is no granulomata formation (original magnification × 100).

**Figure 2:**
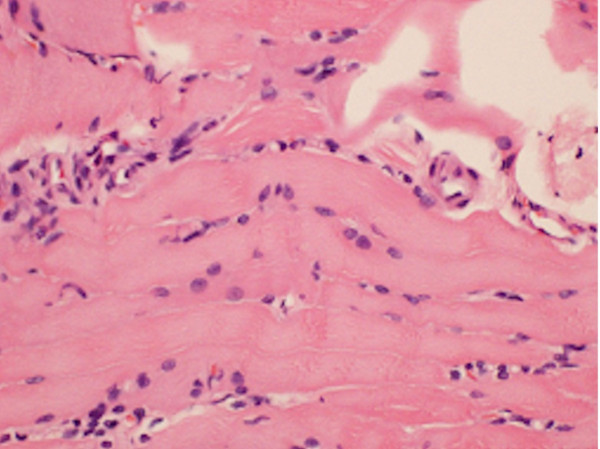
**Section of muscle biopsy stained with HE.** Loss of cross striations, focal disintegration of the sarcoplasm, nuclear vesiculations, and centralization are suggestive of myositis (original magnification × 100).

Toxicology screening of the blood was negative for cocaine, morphine, cannabinoids, amphetamines, barbiturates, benzodiazepines, and tricyclic antidepressants. The blood alcohol estimation was negative and no electrolyte imbalance was detected. Screenings for the antibodies of infective illnesses with similar clinical presentations, such as leptospirosis, dengue, and hepatitis, were negative. Based on the clinical suspicion of an unnoticed snake bite, the patient was treated with poly anti-snake venom, which is active against the bites of cobra, common krait, Sri Lankan krait, and saw-scaled viper. However, no clinical improvement occurred. Septic screening was repeatedly negative despite an ongoing low-grade fever. In the absence of another etiology, the above findings were supportive of an autoimmune origin for the presentation. Electromyography confirmed generalized polymyositis with predominant lower limb involvement. However, anti-nuclear antibody, anti-Jo antibody, and extractable nuclear antigen profiles were negative. Muscle biopsy and histology revealed myositis (Figures 1 and 2) with probable immune pathogenicity.

**Figure 3:**
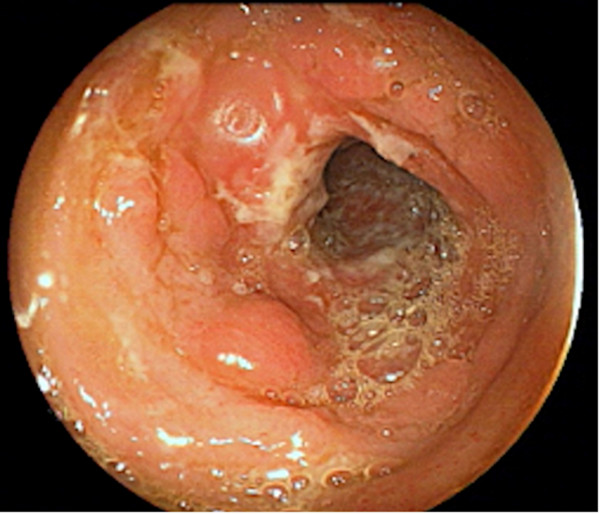
**Endoscopic view of the ascending colon.** The mucosal features of a colon with a cobblestone appearance and surrounding erythema are supportive of a diagnosis of colonic CD.

**Figure 4:**
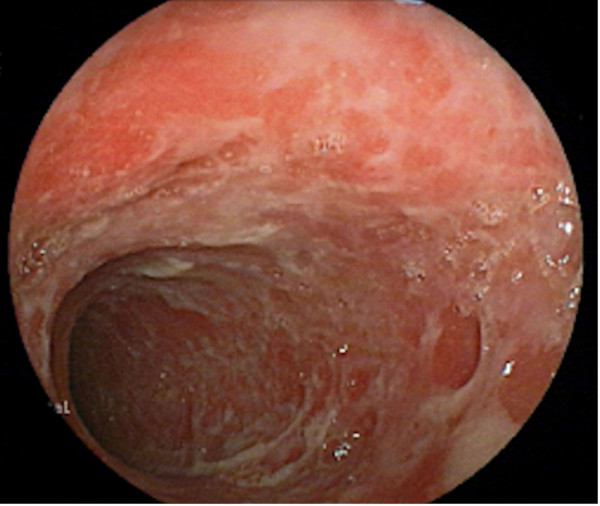
**Endoscopic view of the descending colon.** Multiple, irregular, extensive and superficial ulceration of the colonic mucosa is evident. Normal intervening segments were not seen in this view.

**Figure 5:**
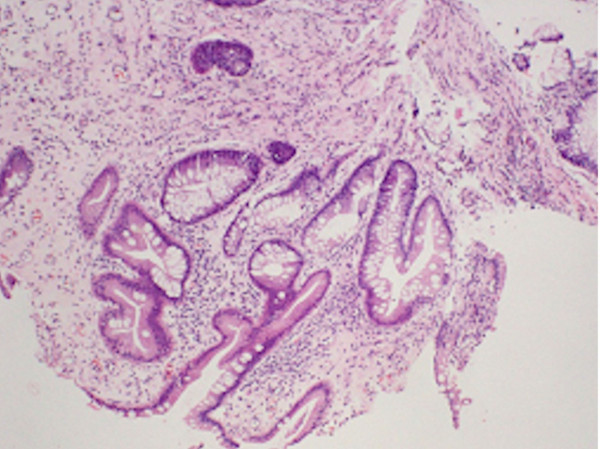
**Section of ileal mucosal biopsy stained with HE showing focal surface ulceration.** The crypt architecture is distorted (non-parallel crypts, variable diameters, and crypt shortening) in conjunction with focal chronic inflammation. Glandular distortion and branching are also presented, which are suggestive of CD (original magnification × 100).

**Figure 6:**
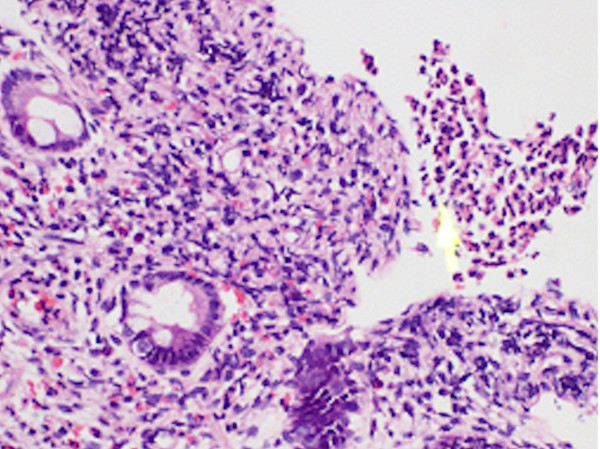
**Section of sigmoid colonic mucosal biopsy stained with HE showing dilated vascular spaces in the focally edematous lamina propria.** There is moderate to dense inflammatory cell infiltrate predominantly composed of lymphocytes and plasma cells with micro-abscess formation (original magnification × 100).

The patient was started on a daily dose of 0.75 mg/kg (45 mg) oral prednisolone with 2 mg/kg (120 mg) azathioprine, and regular renal replacement therapy was carried out together with medical management to support his renal functions. This was followed by a rapid clinical response with significant improvement of muscle weakness. However, he continued to have intermittent diarrhea with blood and mucous that was initially considered part of his acute illness, but later raised the suspicion of an infective or inflammatory etiology. Based on his prolonged stay at the intensive care unit and treatment with broad spectrum antibiotics, a healthcare-associated infection or pseudomembranous colitis was suspected. However, infective screening and *Clostridium difficile* toxins were negative. Ileocolonoscopy revealed extensive ileocolonic aphthous ulceration (Figures 3 and 4), and the histology of the ileum and colon was suggestive of Crohn’s ileocolitis (Figures 5 and 6) based on the European Crohn’s and Colitis Organisation guidelines. Esophagogastroduodenoscopy showed mild antral gastritis, and no radiological contract studies were performed because of the presence of AKI. Amoebiasis and intestinal tuberculosis were excluded by parasitic and microbiological evaluation of mucosal samples. No other extraintestinal manifestations were found, with the exception of sensory motor axonal peripheral neuropathy, which was confirmed by nerve conduction studies. Serological studies evaluating perinuclear anti-neutrophil cytoplasmic and anti-saccharomyces cerevisiae antibodies were negative.

Once the diagnosis of inflammatory polymyositis and CD was established, prednisolone and azathioprine doses were increased to 1 mg/kg (60 mg) daily and 2.5 mg/kg (150 mg), respectively, as the bowel symptoms of the patient continued while on therapy for myositis. Mesacol® (mesalamine), 400 mg daily, and the probiotic Florastor, 250 mg twice daily, were added to the treatment regimen at the same time. Metronidazole was not added in the presence of neuropathy and in the absence of septic complications. The patient achieved clinical remission in both conditions, which was evidenced by the settling of diarrhea and fever, improvement of muscle strength, and significant weight gain by the third week of treatment. Corticosteroid treatment was reduced by 10 mg twice weekly once the endoscopic remission of CD was confirmed. Mesalamine (mesalazine) and Florastor were discontinued and the patient was kept on an azathioprine maintenance dose of 150 mg daily one month after starting treatment. Special attention was given to the patient’s nutrition as his muscles were affected by both the disease process and poor nutrition. A gradual improvement of renal function was followed by an improvement of myositis, which was attributed to the recovery of acute tubular necrosis, secondary to heavy myoglobinuria.

## Conclusions

CD is known to have different immunopathogenic mechanisms; therefore, although the serum markers were negative, an autoimmune disorder remained the most likely possibility in this patient. The observed rapid response to corticosteroids and immunosuppressants also favored this hypothesis. However, the concurrent presentation of two different relapsing inflammatory disorders in a male patient with no family history is unusual. Cuoco previously reported significantly increased blood tumor necrosis factor-α and sphingosine levels caused by enhanced lipopolysaccharide concentration (*p* < 0.01) resulting from altered gut permeability (*p* < 0.01) causing a reduction in muscle fiber size [16]. We suggest that a similar mechanism contributed to the development of an immune response against skeletal muscle in the present case.

Myositis is considered a rare extraintestinal manifestation of CD, but may in fact be a more common occurrence than is reported in patients with IBD. A suggestion for physicians to carefully monitor muscle pain and serum creatine kinase (CK) levels in patients with IBD was previously made by Al-Kawas [12]. Moreover, myositis should be strongly suspected in CD patients with muscle weakness and elevated CK levels, which may help in the early diagnosis and appropriate treatment of CD. Immune-mediated myositis should be considered as an important differential diagnosis for myopathy occurring in IBD secondary to hypokalemia or corticosteroid treatment [17]. In this case, the acute phase of myositis preceded the symptoms of Crohn’s colitis, revealing it to be a separate entity with an inflammatory origin compared with hypokalemia or corticosteroid-induced myopathy.

The immune-mediated pathogenesis of both conditions contributed to their rapid remission following immunosuppressant treatment in our patient. Another study showed that when myositis in CD is immune mediated, the treatment of bowel inflammation should be emphasized as opposed to corticosteroid or other immunosuppressive therapy [10]. In this case, complete recovery of both myositis and CD was achieved with a combination of prednisolone, azathioprine, and mesalamine treatment. The specific effect of mesalamine in this patient cannot be assessed as it was added while increasing the doses of prednisolone and azathioprine.

Myositis and CD together cause profound effects on muscle biology because of their immune-mediateddamage, and nutritional and metabolic deficiencies. Therefore, overall patient management should give equal emphasis to achieving remission as well as correcting metabolic derangements and nutrition. In addition, hypokalemia should be closely monitored and corticosteroids should be used cautiously in this unique association as they can contribute to further muscle injury.

The occurrence of immune polymyositis in CD has a probable related pathophysiological mechanism. Alterations of bowel mucosal permeability causing disruption of the gut-immune barrier may have triggered an immune response against skeletal muscle, which warrants further investigations. Standard immune modulators used in CD such as prednisolone and azathioprine are effective first-line therapies to induce remission in both conditions. Immune-mediated myositis is an important differential diagnosis for hypokalemia or corticosteroid-induced myopathy, and muscle biopsy plays an important role in its differentiation. This case provides new insights into disease pathogenesis and useful evidence for the management of CD associated with immune polymyositis.

### Consent

Written informed consent was obtained from the patient for publication of this case report and any accompanying images. A copy of the written consent is available for review by the Editor of this journal.

## Abbreviations

CD: Crohn’s disease; IBD: Inflammatory bowel disease; AKI: Acute kidney injury; ALT: Alanine transaminase; HE: Hematoxylin and eosin stain; CK: Creatine kinase.

## Competing interests

The authors declare that they have no competing interests.

## Authors’ contributions

VRB participated in the management of the patient and drafted the manuscript. NF carried out the endoscopies and involved in the management of the patient. UL participated in the management and clinical decision making. CJ participated in coordination and helped to draft the manuscript. All authors read and approved the final manuscript.

## Authors’ information

VRB: (MBBS, pursuing MD (medicine)). Registrar in medicine, National Hospital, Colombo, Sri Lanka.

NF: (MBBS, MD (medicine), MRCP). Senior registrar in gastroenterology, National Hospital, Colombo, Sri Lanka.

UL: (MBBS, MD (medicine), MRCP, FRACP). Senior registrar in medicine, Sri Jayawardanapura General Hospital, Colombo, Sri Lanka.

CJ: (MBBS, MD (medicine)). Consultant physician, Sri Jayawardanapura General Hospital, Colombo, Sri Lanka.

## Pre-publication history

The pre-publication history for this paper can be accessed here:

http://www.biomedcentral.com/1471-230X/14/35/prepub
